# Highly efficient CRISPR/Cas9‐mediated exon skipping for recessive dystrophic epidermolysis bullosa

**DOI:** 10.1002/btm2.10640

**Published:** 2024-01-17

**Authors:** Alex du Rand, John Hunt, Christopher Samson, Evert Loef, Chloe Malhi, Sarah Meidinger, Chun‐Jen Jennifer Chen, Ashley Nutsford, John Taylor, Rod Dunbar, Diana Purvis, Vaughan Feisst, Hilary Sheppard

**Affiliations:** ^1^ School of Biological Sciences The University of Auckland Auckland New Zealand; ^2^ Te Whatu Ora Health New Zealand Te Toka Tumai Auckland New Zealand

**Keywords:** CRISPR/Cas9, exon skipping, gene therapy, recessive dystrophic epidermolysis bullosa (RDEB), ribonucleoproteins, structural variants

## Abstract

Gene therapy based on the CRISPR/Cas9 system has emerged as a promising strategy for treating the monogenic fragile skin disorder recessive dystrophic epidermolysis bullosa (RDEB). With this approach problematic wounds could be grafted with gene edited, patient‐specific skin equivalents. Precise gene editing using homology‐directed repair (HDR) is the ultimate goal, however low efficiencies have hindered progress. Reframing strategies based on highly efficient non‐homologous end joining (NHEJ) repair aimed at excising dispensable, mutation‐harboring exons offer a promising alternative approach for restoring the *COL7A1* open reading frame. To this end, we employed an exon skipping strategy using dual single guide RNA (sgRNA)/Cas9 ribonucleoproteins (RNPs) targeted at three novel *COL7A1* exons (31, 68, and 109) containing pathogenic heterozygous mutations, and achieved exon deletion rates of up to 95%. Deletion of exon 31 in both primary human RDEB keratinocytes and fibroblasts resulted in the restoration of type VII collagen (C7), leading to increased cellular adhesion in vitro and accurate C7 deposition at the dermal‐epidermal junction in a 3D skin model. Taken together, we extend the list of *COL7A1* exons amenable to therapeutic deletion. As an incidental finding, we find that long‐read Nanopore sequencing detected large on‐target structural variants comprised of deletions up to >5 kb at a frequency of ~10%. Although this frequency may be acceptable given the high rates of intended editing outcomes, our data demonstrate that standard short‐read sequencing may underestimate the full range of unexpected Cas9‐mediated editing events.

**Translational Impact Statement**

Recessive dystrophic epidermolysis bullosa (RDEB) is a fragile skin condition caused by mutations in the type VII collagen (C7) gene. Skin lesions could be treated with bulk gene corrected, patient‐specific engineered skin equivalents, if gene editing rates were sufficiently high. We describe a highly efficient dual sgRNA/Cas9‐mediated exon skipping strategy targeting C7 in both major skin cell types. We apply this approach to three previously untargeted exons carrying pathogenic mutations, with functionality verified for one exon. We also demonstrate that short‐read sequencing alone is insufficient to comprehensively analyze on‐target edits.

## 1 INTRODUCTION

Epidermolysis bullosa (EB) comprises a heterogeneous group of inherited skin disorders characterized by fragile skin and mucosal membranes. The recessive dystrophic EB (RDEB) subtype is associated with severe symptoms and is caused by biallelic, loss‐of‐function mutations in the *COL7A1* gene encoding the alpha‐1 chain of type VII collagen (C7).^1^ C7 is the main constituent of the large anchoring fibril structures which are critical for providing adhesion between the dermal and epidermal skin layers. Reductions or functional impairments in C7 can therefore lead to the generation of severe and poorly healing blisters following minor mechanical trauma, which can translate into more systemic and life‐threatening complications.^2^

In the absence of a cure, a variety of therapeutic strategies are being investigated including allogeneic fibroblast therapy,^3^ protein replacement therapy (Clinical Trial Identifier #NCT03752905), and a transiently expressed gene therapy.^4^ However, as these approaches fail to address the underlying genetic mutation, they are unable to provide a permanent cure. *Ex vivo* gene therapies offer a promising alternative that may provide lifelong benefits. They include gene replacement strategies based on the addition of healthy copies of defective genes packaged within integrating viral vectors and delivered into patient cells. These have already progressed to clinical trials for some forms of EB with encouraging results.^5^ However, several issues remain, including random genomic integration of the viral vectors, variable transgene expression, and the inability to correct dominant‐negative mutations.^6^ To bypass these issues, recent efforts have focused on site‐specific, designer nucleases capable of introducing genomic modifications at specific loci.^7^, ^8^, ^9^, ^10^, ^11^, ^12^, ^13^, ^14^ Conventional CRISPR‐based nucleases use a single guide RNA (sgRNA) to target a Cas9 nuclease to specific genomic sites where they generate double‐strand breaks (DSBs) that are repaired primarily by either homology‐directed repair (HDR) or non‐homologous end joining (NHEJ).^15^ Although precise mutation correction by means of HDR is preferred, these strategies have generally suffered from suboptimal efficiencies, often necessitating antibiotic selection or the isolation and expansion of desired clonal cells.^16^ In comparison, *COL7A1* reframing approaches exploiting NHEJ‐based repair have enabled reframing efficiencies ranging from 60% to 81%, allowing the use of the entire edited polyclonal cell population.^7^, ^11^, ^13^

The therapeutic removal of several in‐frame exons (including exons 70, 73, 80, and 105) within the large *COL7A1* triple helical domain using antisense oligonucleotides has previously been demonstrated.^17^, ^18^, ^19^ More recently, Bonafont and colleagues used dual sgRNAs in primary RDEB keratinocytes to remove exon 80 of *COL7A1* containing a prevalent frameshift mutation with high efficiency (81%).^7^ In this study, we applied a dual sgRNA/Cas9 reframing strategy to three *COL7A1* exons (31, 68, and 109), which have not previously been targeted for excision, containing heterozygous mutations present in New Zealand patients. We achieved highly efficient exon deletion rates of up to 90% and 95% in primary RDEB fibroblasts and keratinocytes, respectively. Specifically, the deletion of exon 31 in primary RDEB skin cells resulted in the restoration of C7 expression comparable to wild type cells. The restored C7 variant increased skin cell adhesion *in vitro* and was accurately deposited at the dermal‐epidermal junction in a 3D skin model. Comprehensive on‐target analysis using long‐read Nanopore sequencing revealed the presence of large Cas9‐induced structural variants (SVs).

Taken together, we demonstrate that a Cas9 nuclease‐mediated exon skipping approach offers an efficient, alternative repair strategy that avoids the low correction rates reported using many HDR‐based approaches. We extend the range of known *COL7A1* exons that can be removed without losing function and we show that this approach is effective in both primary keratinocytes and dermal fibroblasts. This strategy potentially has broad applicability to a wide range of *COL7A1* exons. However, our findings also emphasize the importance of comprehensively assessing unexpected on‐target editing events.

## 2 RESULTS

### 2.1 Dual sgRNA Cas9 nuclease‐ribonucleoproteins enable highly efficient targeted deletion of *COL7A1* exons in RDEB skin cells

**FIGURE 1:**
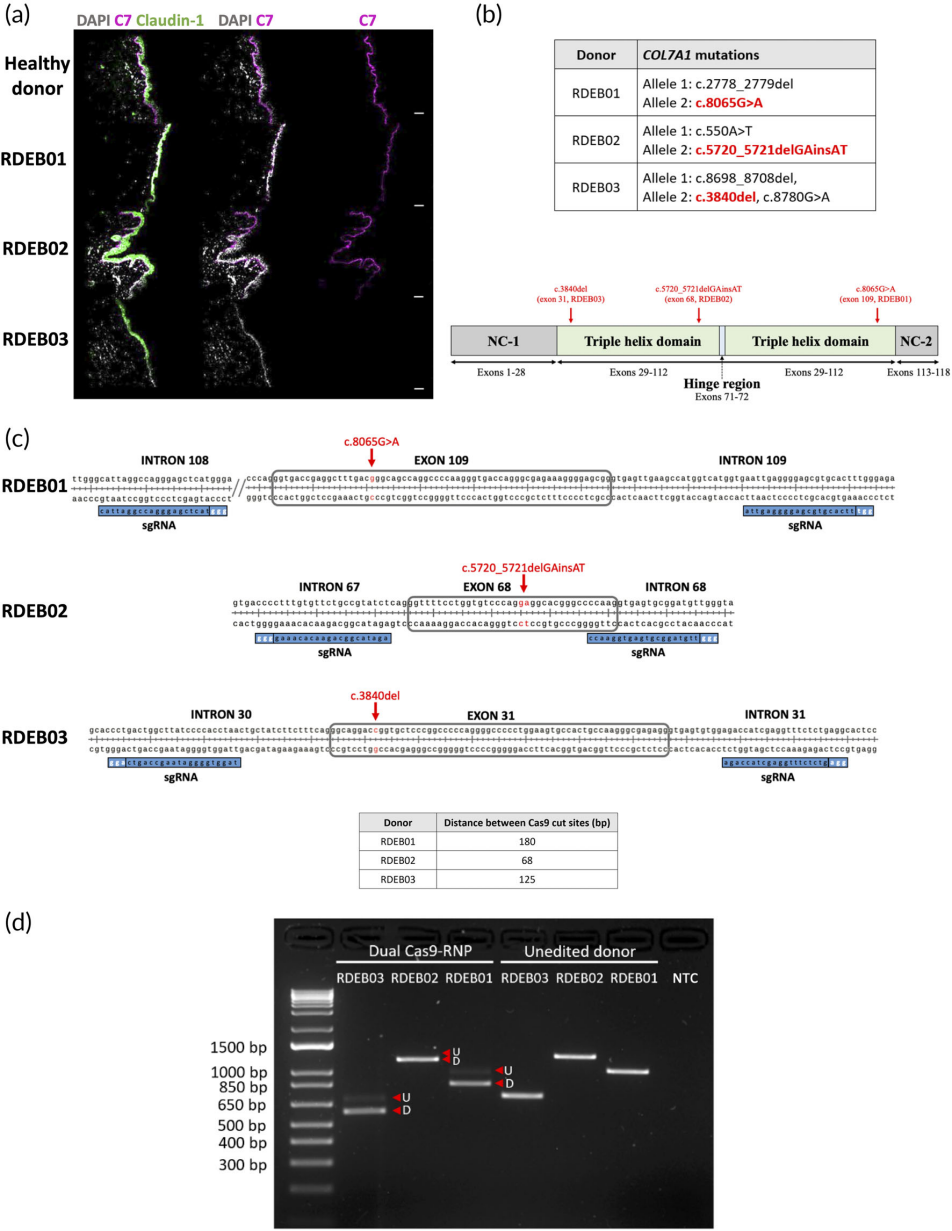
Dual Cas9‐RNP exon skipping strategy for three RDEB donors. (a) Immunofluorescence of skin sections from RDEB01, RDEB02, and RDEB03 comparing C7 (magenta) to a healthy donor. The righthand side panel shows C7 alone (magenta), middle panel shows a merged image of C7 with the nuclear stain DAPI (gray), and the lefthand side panel is a merge of C7, DAPI and the epidermal marker Claudin‐1 (green). Scale bars = 50 μm. (b) The *COL7A1* mutations for each donor are shown with the targeted mutations highlighted in red. The structure of the *COL7A1* gene and the positions of these mutations are also highlighted. The gene is composed of three domains: non‐collagenous domain 1 (NC‐1), triple helix domain (THD), and non‐collagenous domain 2 (NC‐2). The THD contains a “hinge” region which provides the triple helix with flexibility. (c) Design of the dual sgRNAs targeting a different exon for each RDEB donor. The PAM sites for each sgRNA are depicted with white text. Targeted mutations are shown in red. The distance between the Cas9 cut sites for each sgRNA pair is listed in the table. (d) An agarose gel analysis of DNA amplicons spanning the exon of interest following dual Cas9 exon skipping in keratinocytes from each RDEB donor. Lower molecular weight bands represent alleles with the target exon deleted (“D”) and the higher molecular weight bands represent undeleted alleles (“U”). NTC, no template control (H_2_O).

Three RDEB donors (hereafter referred to as RDEB01, RDEB02, and RDEB03) harboring compound heterozygous mutations in the *COL7A1* gene were enrolled in this study. Immunofluorescence of skin sections from each donor revealed variable C7 expression compared to a healthy control (Figure 1a). This likely reflects the heterogeneous nature of the mutations which can affect protein expression or function. In each donor, we targeted a predicted pathogenic mutation contained within an in‐frame, triple helical domain exon (based on the *COL7A1* reference transcript, accession number NM_000094.3) (Figure 1b). To achieve this, we designed dual sgRNAs targeted to the introns flanking each mutation (Figure 1c, Table S1). In the absence of donor repair templates, NHEJ repair was predicted to ligate the breakpoints to generate deletions spanning the entirety of each exon to generate a functional chimeric intron, without disrupting the *COL7A1* open reading frame.

We electroporated the sgRNAs and Cas9 into the cells as ribonucleoprotein (RNP) complexes. Compared to other delivery methods, RNPs are associated with higher gene disruption efficiencies, lower off‐target genotoxicity due to their short cellular half‐life, and avoidance of undesired recombination events caused by exogenous DNA delivery constructs.^20^, ^21^ We edited primary RDEB keratinocytes from all three donors, but focused further studies on cells derived from donor RDEB03 due to their null C7 phenotype which would facilitate downstream analysis of *COL7A1* reframing (i.e., restoration of transcription and protein expression could be more easily identified). Following electroporation, we extracted genomic DNA and used polymerase chain reaction (PCR) to amplify regions spanning 698 bp to 1203 bp over each exon (Table S2). The presence of lower molecular weight agarose gel bands of the expected sizes indicated highly efficient deletion of all three exons (Figure 1d).

**FIGURE 2:**
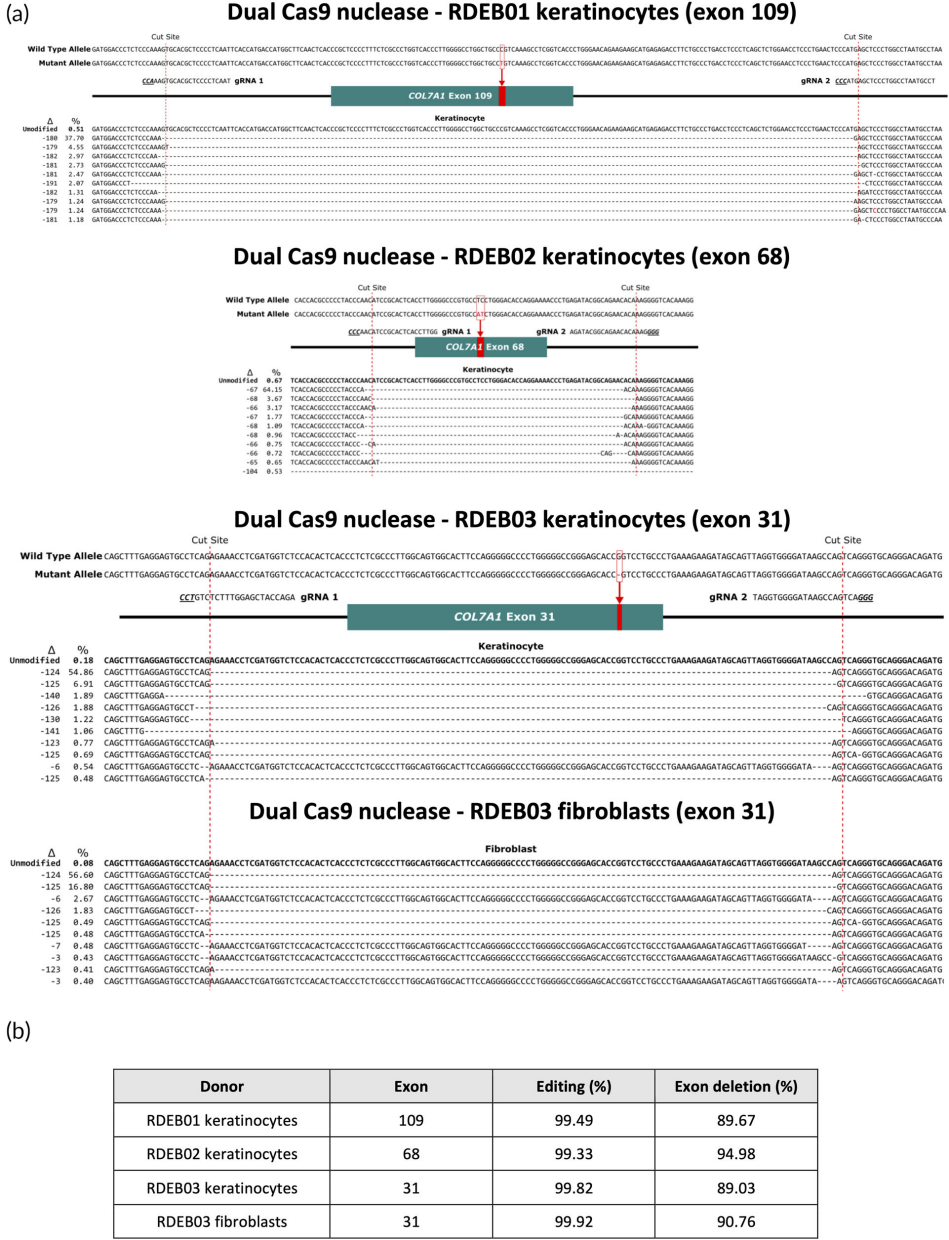
Nanopore sequencing analysis of the editing outcomes resulting from dual Cas9‐RNP exon skipping. (a) Diagrammatic representation of the 10 most common deletion events resulting from dual Cas9 nuclease‐RNP editing in RDEB skin cells. Both the wild type allele and the mutant allele, with the mutation highlighted in red, are shown for each donor. The position of the sgRNAs is also shown, with the PAM sites underlined. The predicted Cas9 cut sites are depicted with red dotted lines. For each of the edited alleles, the allele frequency and the deletion size are shown. (b) Summary of the total editing and exon deletion efficiencies for each skin cell donor using dual Cas9 nuclease‐RNPs.

To precisely characterize the repair events generated at the on‐target sites, PCR amplicons derived from the bulk‐edited cell populations were subject to Nanopore sequencing, and the resulting sequence reads were analyzed using CRISPResso2 (http://crispresso2.pinellolab.org). A variety of repair outcomes were uncovered by this analysis (Figure 2a). In keratinocytes, for RDEB01 (23,435 analyzed reads), 37.7% of repair outcomes corresponded to the predicted deletion of exon 109 spanning the Cas9 cut sites. For RDEB02 (3216 analyzed reads), the predominant repair event accounting for 64.15% of alleles was characterized by the predicted deletion of exon 68 plus the insertion of an A (Figure 2a). Similarly, for RDEB03 the predominant repair event, accounting for 54.86% in keratinocytes (53,966 analyzed reads) and 56.6% in fibroblasts (39,454 analyzed reads), corresponded to the predicted deletion of exon 31 with an AG insertion (Figure 2a). Additionally, a range of other repair events spanning the target exons were also detected for all donors. While these repair outcomes generated insertions and deletions (INDELs), they were mostly small (1–5 bp) and therefore unlikely to perturb the splicing of the newly generated chimeric intron. Overall, the total proportion of alleles with a deletion spanning the target exon (but not additional exons) was 89.67% for RDEB01, 94.98% for RDEB02, 89.03% for RDEB03 keratinocytes, and 90.76% for RDEB03 fibroblasts (Figure 2b). After accounting for the small proportion of remaining editing events corresponding to small INDELs at the individual cut sites, ~99% of alleles for all donors showed evidence of on‐target editing (Figure 2b). Proliferation analysis revealed that RDEB03 keratinocytes had comparable rates of proliferation following Cas9‐RNP editing compared to untreated and wild type keratinocytes over a four week period, confirming that our editing protocol and cell culture system did not have a detrimental effect on cell growth (Figure S2a,b).

Lastly, using the same sgRNA pairs, we tested a dual nickase exon skipping approach using the D10A variant, which is associated with a reduced risk of off‐target effects.^16^ Nanopore sequencing analysis revealed editing and targeted exon deletion rates of up to 80% and 23%, respectively (Figure S3). However, dual‐nicking generated a diverse pool of deletion events that precluded protein re‐expression (data not shown). Therefore, we did not pursue this strategy further.

### 2.2 Dual Cas9‐RNP exon skipping does not lead to detectable off‐target INDEL formation but does generate large on‐target structural variants

To assess the safety of dual Cas9 nuclease‐RNP exon skipping we analyzed the top in silico predicted off‐target sites. Genomic DNA extracted from bulk‐edited RDEB03 keratinocytes and fibroblasts was used to generate amplicons ranging from 900−1900 bp spanning 12 off‐target sites (six per sgRNA) (Table S2). Amplicons were Nanopore sequenced, and the reads analyzed using CRISPResso2 (minimum 5000 reads analyzed per site). This revealed no evidence of off‐target editing, as none of the differences between the edited and unedited samples met the threshold for significance set by CRISPResso2 (Bonferroni corrected *p*‐value = 1, Table S3).

**FIGURE 3:**
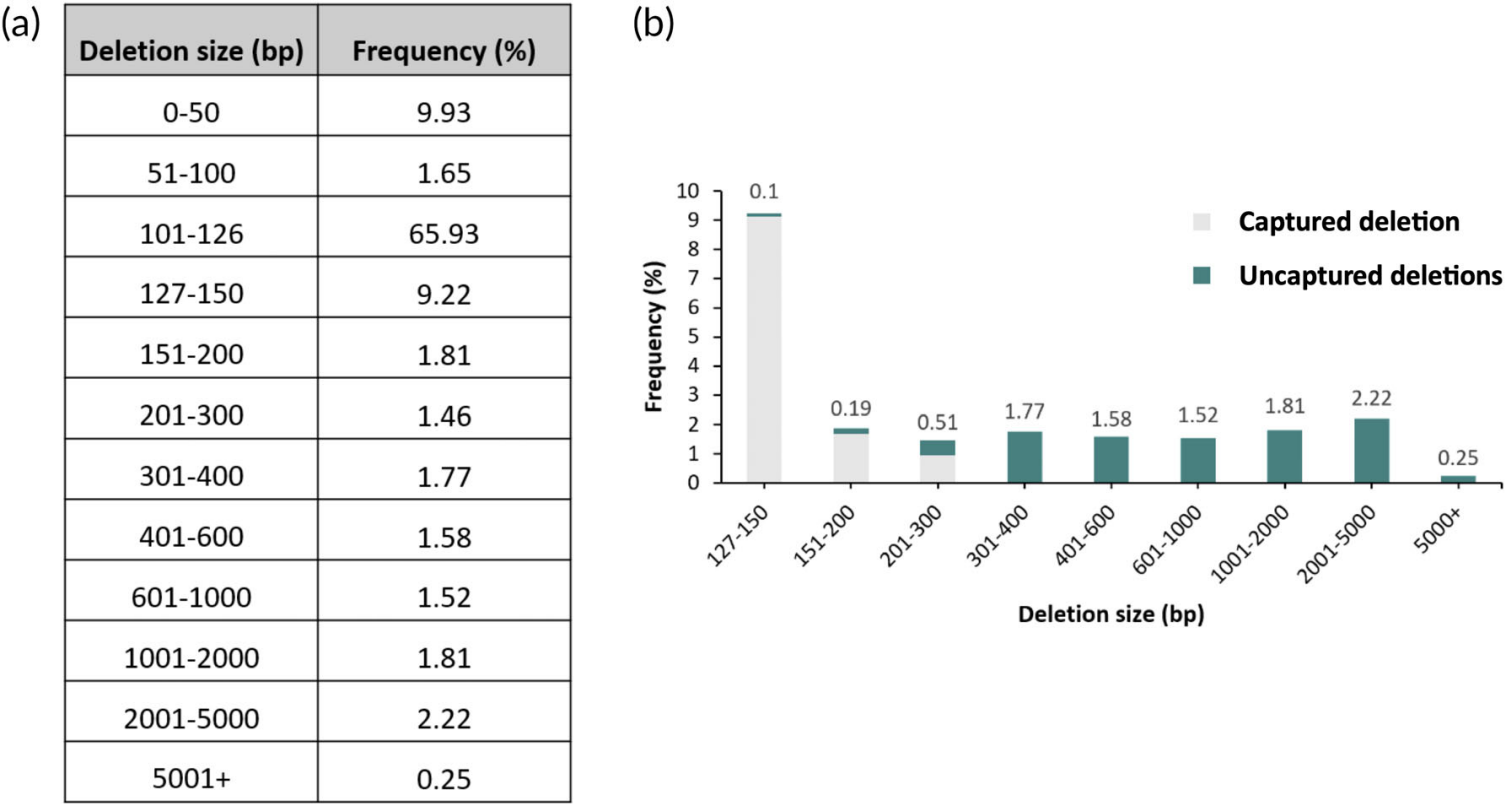
Long‐range (10 kb) Nanopore sequencing of on‐target editing outcomes resulting from dual Cas9‐RNPs targeting exon 31 in RDEB03 keratinocytes. (a) The frequency and size of detected deletions resulting from dual Cas9‐RNP editing. (b) Graphical representation of the frequency of deletion events >126 bp (as a percentage of total reads) that were captured (gray) or not (green) by short amplicon genotyping.

To more comprehensively assess the safety profile of dual Cas9‐RNP exon skipping, we Nanopore sequenced 10 kb amplicons centered over exon 31 using genomic DNA extracted from bulk‐edited RDEB03 keratinocytes (3142 analyzed reads, primers listed in Table S2). This analysis revealed the presence of a range of larger deletion events, including deletions between 200 and 1000 bp (6.36% of total alleles), deletions between 1 and 5 kb bp (4.28% of total alleles), and a small proportion of deletions >5 kb (0.25% of total alleles) (Figure 3a). To determine the proportion of these events that evaded detection by short amplicon genotyping we first calculated the number of long‐range sequencing reads missing at least one of the primer sites required for short‐range amplification. This identified that 8.62% of long‐range reads had a deletion that ablated at least one primer site. To assess whether there were any additional deletion events not captured by short amplicon sequencing we directly compared the long‐range and short‐range sequencing data. This revealed an additional small pool of deletions (~1%) that failed to be captured by short‐range sequencing even though both primer sites remained intact. We found that these deletions would have resulted in the generation of short amplicons that were likely lost during processing. Taken together, a total of 9.95% of deletions were found to have initially evaded detection by short amplicon genotyping (Figure 3b). After accounting for these events, the revised exon 31 deletion efficiency (i.e., deletions only spanning the target exon) according to the long‐range sequencing data was 78%.

### 2.3 Deletion of exon 31 in RDEB03 skin cells leads to predicted mRNA splicing and increases C7 transcription

**FIGURE 4:**
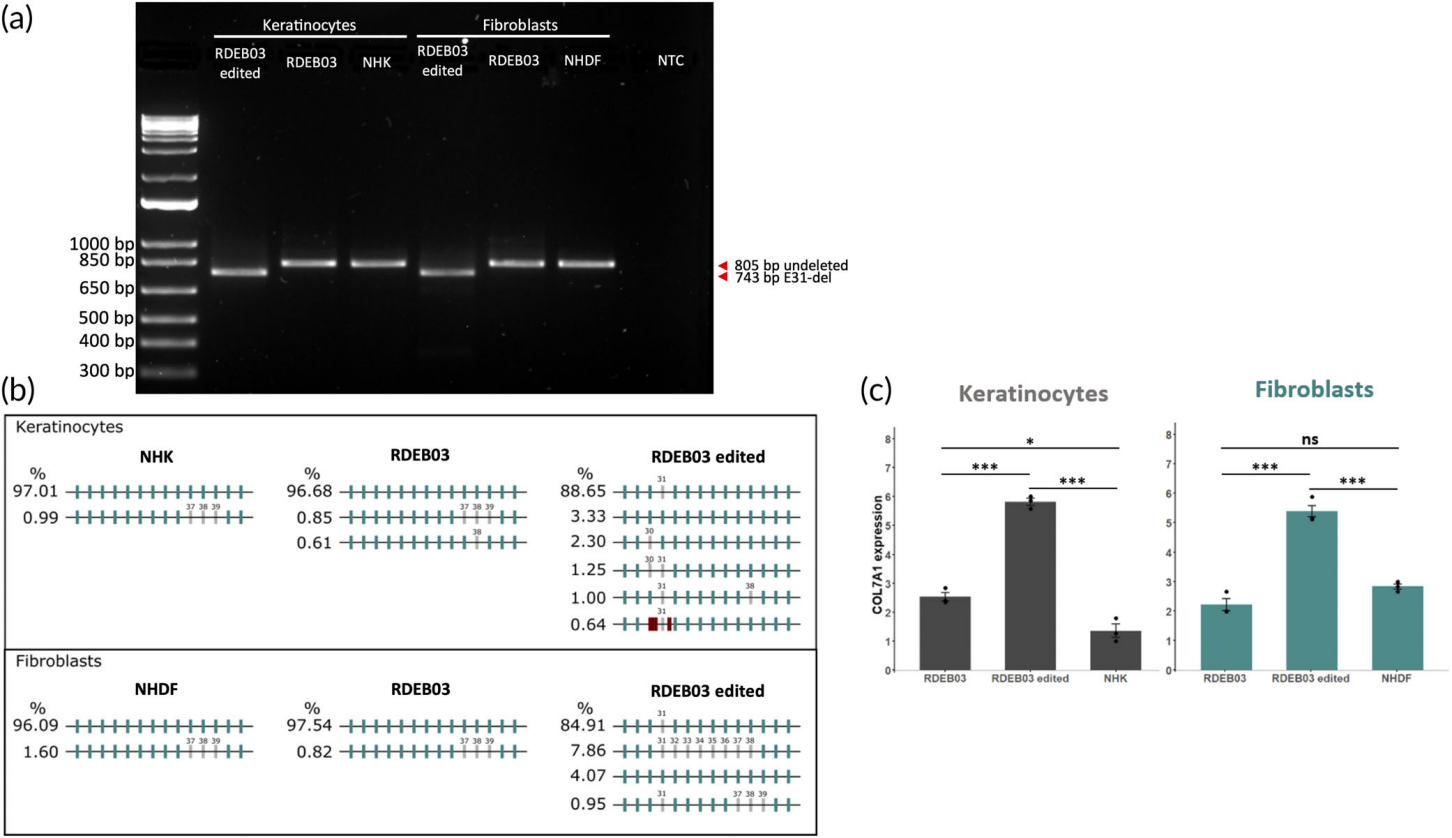
*COL7A1* transcription analysis of exon skipped RDEB03 keratinocytes and fibroblasts. (a) RT‐PCR agarose gel analysis of amplicons derived from exons 28–41 of *COL7A1* transcripts. An 805 bp unmodified band is present in all samples. In the edited samples, a lower molecular weight band of 743 bp is also present and represents transcripts lacking exon 31. NHK, normal human keratinocytes; NHDF, normal human dermal fibroblasts; NTC, no template control. (b) Characterization of the most prevalent *COL7A1* transcripts in RDEB03 skin cells by Nanopore sequencing analysis. Exons and introns are depicted by thick green bars and thin intervening gray lines, respectively. Exons missing from the transcript are depicted in gray with their corresponding number shown. Red bars represent intron read‐through events whereby part of an intron has been spliced into the final transcript. (c) Quantification of full‐length *COL7A1* mRNA by ddPCR using a primer set targeting exon 109 of *COL7A1*. Three individual experiments were performed for keratinocytes and one experiment for fibroblasts, *n* = 3. The data represent the mean ± SEM; *p*‐value <0.05 (*); *p*‐value <0.01 (**); *p*‐value <0.001 (***). ns, no significance.

We designed the dual Cas9‐RNP exon 31 deletion strategy to generate a chimeric intron following NHEJ, with a newly defined donor and acceptor splicing motif from introns 30 and 31. However, the possibility of activating nearby cryptic splice sites following the removal of authentic splice sites could lead to the generation of aberrantly spliced transcripts. To confirm bona fide restoration of the *COL7A1* reading frame corresponding to the generation of transcripts with precise in‐frame joining at the exon 30–32 border, we examined *COL7A1* splicing. To this end, we performed reverse transcriptase PCR (RT‐PCR) by amplifying an 805 bp region spanning exons 28–41 of *COL7A1* mRNA (see Table S2 for primers). Following agarose gel electrophoresis, molecular weight bands of the predicted size were observed in the edited samples, indicating that exon 31 had been deleted from the majority of transcripts (Figure 4a). To precisely characterize splicing events, amplicons were subject to Nanopore sequencing (minimum analyzed reads = 40,000, Table S4). This analysis verified that 88.65% of transcripts in bulk‐edited RDEB03 keratinocytes and 84.91% of transcripts in bulk‐edited RDEB03 fibroblasts had been spliced as predicted with the targeted removal of exon 31 (Figure 4b). A smaller proportion of transcripts (4.71% for RDEB03 keratinocytes and 8.49% for RDEB03 fibroblasts) showed different splicing patterns. This included the deletion of additional exons other than exon 31 (4.07% for RDEB03 keratinocytes and 8.20% for RDEB03 fibroblasts), or the inclusion of intronic sequences in the final transcript (0.64% for RDEB03 keratinocytes and 0.29% for RDEB03 fibroblasts) (Figure 4b, Table S5). Additionally, naturally occurring alternatively spliced transcripts were detected at low levels, including one lacking exon 37–39 and another lacking exon 38 (Figure 4b).

Next, we used Droplet Digital PCR (ddPCR) to quantify *COL7A1* mRNA in bulk‐edited RDEB03 keratinocytes and fibroblasts (see Table S2 for primers). Interestingly, this analysis revealed elevated levels of *COL7A1* transcription in untreated RDEB03 keratinocytes compared to a healthy control (Figure 4c). This finding might be explained by prior research showing that lack of C7 can compromise trafficking of large ECM proteins and lead to their intracellular accumulation.^22^ This buildup results in endoplasmic reticulum stress and consequently an induction of TGF‐β signaling, which is known to upregulate *COL7A1* transcription.^22^ Following the deletion of exon 31, a further increase in transcription was detected in RDEB03 keratinocytes. Similarly, an increase in *COL7A1* transcription was detected following exon 31 deletion in RDEB03 fibroblasts (Figure 4c). Taken together, these data demonstrate that dual Cas9‐RNP‐mediated removal of exon 31 containing a pathogenic frameshift mutation leads to precise in‐frame joining at the border of exons 30–32 in the majority of transcripts, resulting in an increase in C7 transcription.

### 2.4 The deletion of exons 31 restores C7 protein expression in RDEB03 keratinocytes

**FIGURE 5:**
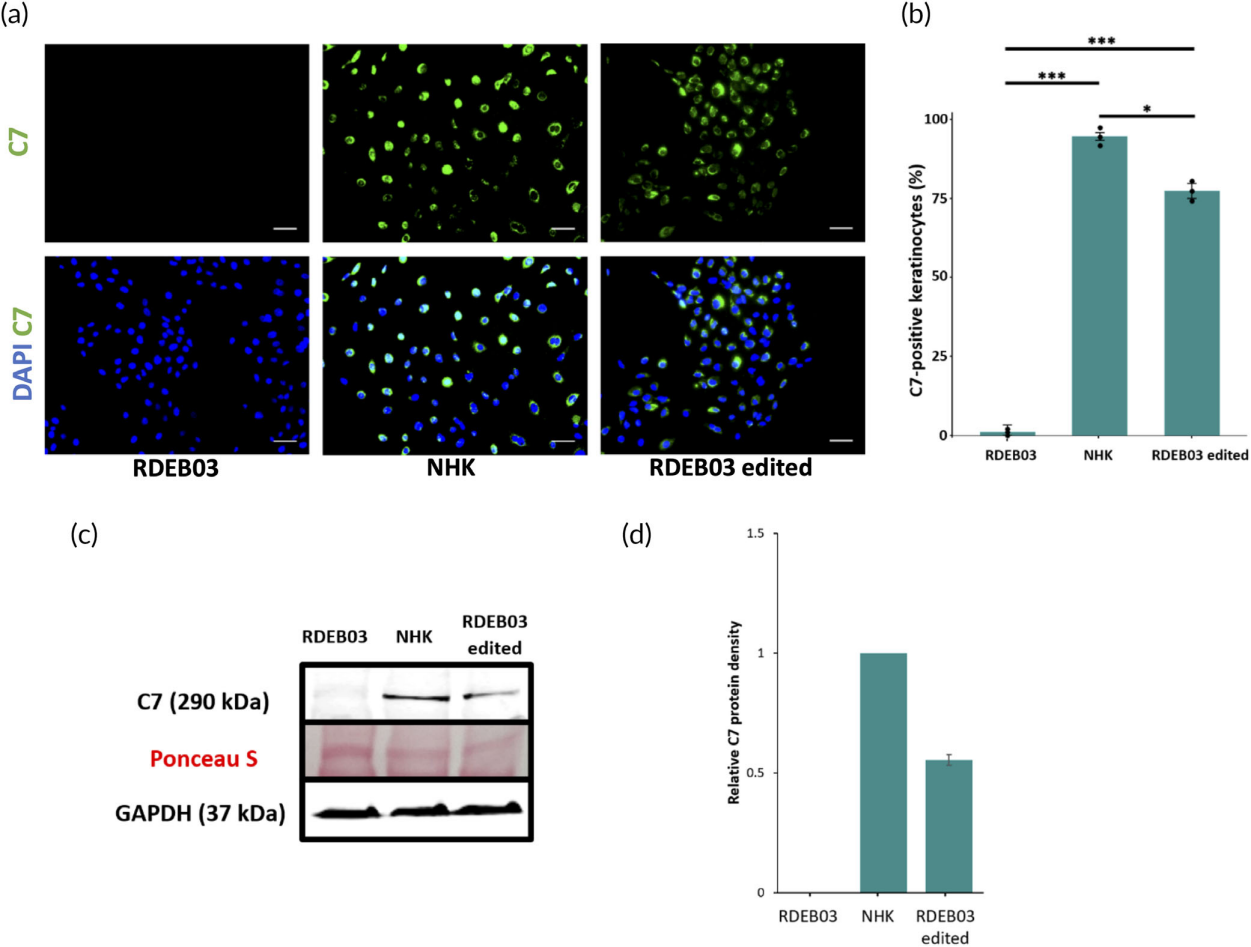
Analysis of C7 protein in exon skipped RDEB03 keratinocytes and wild type keratinocytes following dual Cas9‐RNP editing. (a) Immunofluorescence of C7 in RDEB03 keratinocytes before and after exon 31 deletion compared to a healthy control. C7 is shown in green and DAPI in blue. Scale bars = 50 μm. (b) Quantification of C7‐positive keratinocytes. Three individual experiments were performed, *n* = 3. The data represent the mean ± SEM; *p*‐value <0.05 (*); *p*‐value <0.01 (**); *p*‐value <0.001 (***). NHK, normal human keratinocytes. (c) Analysis of C7 expression by western blot using cell lysates from edited and unedited RDEB03 keratinocytes compared to NHK. Ponceau S staining of the membrane was used to assess protein loading and the housekeeper gene GAPDH was used as a loading control. (d) Quantification of C7 western blot bands normalized to the density of GAPDH and expressed relative to the density of NHK. The data represent the mean ± SEM of two individual experiments.

Next, we investigated whether the excision of exon 31 harboring a pathogenic frameshift mutation restored the expression of C7 protein in RDEB03 keratinocytes, the primary C7‐producing cell type in human skin. Immunofluorescence of bulk‐edited RDEB03 keratinocytes revealed that a high proportion of the polyclonal cell population (~80%) expressed C7 (Figure 5a,b, Figure S1). Western blot analysis of cell lysates showed C7 restoration equating to approximately half (57%) the level of wild type keratinocytes, as expected given the correction of only the mutant allele (Figure 5c,d). The size of the C7 protein detected in the wild type and the RDEB03 edited samples was approximately the same (290 kDa) (Figure 5c). This was expected given that the small 21 amino acid difference between wild type C7 and the C7 variant lacking exon 31 could not be resolved at this molecular weight. Taken together, these data confirmed restoration of the *COL7A1* open reading frame following the removal of the pathogenic mutation within exon 31.

Given that RDEB01 and RDEB02 skin cells express C7 protein similar to wild type levels (Figure 1a), we were unable to use immunofluorescence or western blot analysis to assess the impact of deleting exons 68 (RDEB02) or 109 (RDEB01) on the *COL7A1* open reading frame. To work around this, we performed immunofluorescence on wild type keratinocytes to assess whether the removal of these exons would disrupt normal C7 protein expression. This analysis revealed no significant difference in the number of C7‐expressing keratinocytes between the unedited and dual Cas9‐RNP edited samples containing a deletion of exon 68 or 109 in 95% and 90% of cells, respectively, indicating that the open reading frame had been maintained following the removal of each individual exon (Figure S4a,b).

### 2.5 Exon 31 deletion increases RDEB03 fibroblast adhesion *in vitro* and restores normal C7 deposition in a 3D skin model

**FIGURE 6:**
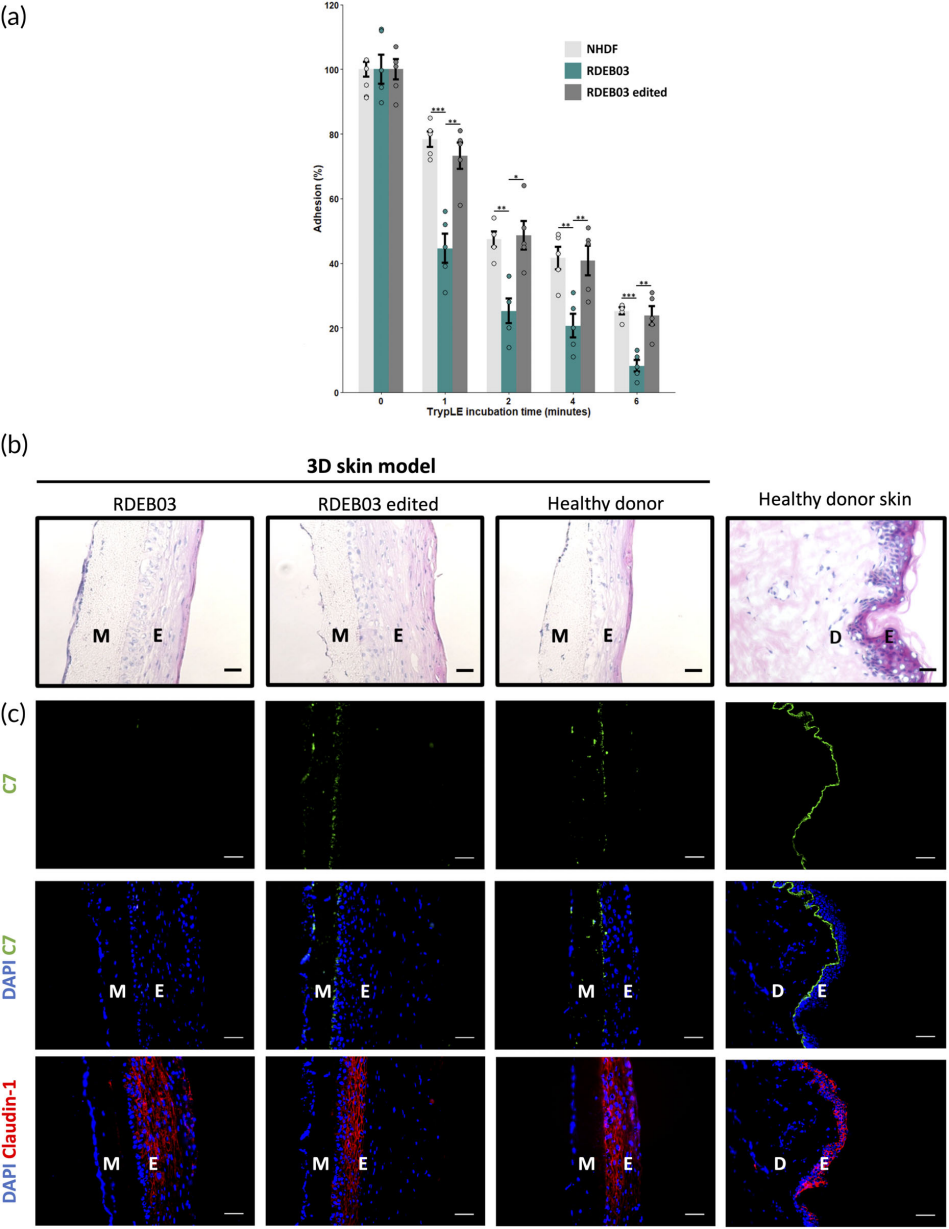
Functional analysis of the restored C7 variant in exon 31 skipped RDEB03 fibroblasts and keratinocytes. (a) *In vitro* adhesion assay of exon skipped RDEB03 fibroblasts using the TrypLE dissociation reagent. Cell adhesion is measured as the proportion of fibroblasts adhered to cell culture vessels after the specified incubation times with TrypLE. Two independent experiments were performed, *n* = 5. The data represent the mean ± SEM; *p*‐value <0.05 (*); *p*‐value <0.01 (**); *p*‐value <0.001 (***). NHDF, normal human dermal fibroblasts. H&E staining (b) or immunofluorescence (c) of a skin model using RDEB03 skin cells (left), exon skipped RDEB03 skin cells (middle left) and healthy skin cells (middle right). A skin section from a healthy donor is also included (right) with the epidermal (E) and dermal (D) layers shown. The PLGA mesh containing fibroblasts (M) and stratified epidermal layer (E) are indicated for the skin model. C7 staining by immunofluorescence is shown in green and the epidermal marker Claudin‐1 in red. DAPI is shown in blue as a nuclear stain. Scale bars = 50 μm.

Previous research has shown that RDEB fibroblasts exhibit poor adhesion properties *in vitro* compared to wild type cells due to a lack of C7.^23^, ^24^, ^25^ It was demonstrated that the adhesion capabilities of RDEB fibroblasts could be restored following genetic correction of *COL7A1* or retroviral transduction with wild type *COL7A1*. Therefore, to determine whether the C7 variant lacking exon 31 increased the adhesion of RDEB03 fibroblasts we conducted an *in vitro* adhesion assay. Prior to correction, RDEB03 fibroblasts displayed poor adhesion to cell culture vessels. Adhesion significantly increased post editing and was similar to wild type cell adhesion capabilities (Figure 6a). These data suggest that the C7 variant lacking exon 31 was capable of trimerizing (a requirement for C7 secretion) and subsequent secretion.

Next, we used an in‐house 3D skin model (patent # WO2019066662A1, WO2019066664A1, WO2018124887A1) to determine whether expression of the C7 protein variant localized correctly to the basement membrane. To generate this model we seeded keratinocytes on an electrospun poly(lactic‐co‐glycolic acid) PLGA mesh, a material already in clinical use for the production of absorbable sutures,^26^ coated with type I collagen and infiltrated with fibroblasts to provide trophic support for keratinocyte growth. We cultured the 3D skin model for 3 weeks at an air‐liquid interface to allow the keratinocytes to generate a stratified epithelium on the mesh (Figure 6b). As expected, no C7 was detected by immunofluorescent analysis of our 3D skin model generated using unedited RDEB03 skin cells (Figure 6c). In contrast, incorporation of bulk‐edited RDEB03 keratinocytes and fibroblasts into this model confirmed that both cell types expressed the C7 variant. Although C7 staining was slightly discontinuous, C7 expression within the stratified epidermis was restricted to the basal layer at the basement membrane, consistent with the expression pattern of C7 observed in human skin (Figure 6c).

## 3 DISCUSSION

Engineered CRISPR/Cas9 gene‐edited, patient‐specific skin equivalents represent a promising strategy to treat problematic wounds for people with EB. This approach relies on *ex vivo* gene editing of skin cells. HDR‐mediated gene editing of skin cells is generally limited by low editing rates, so an NHEJ‐mediated exon skipping approach is a promising, efficient alternative. We demonstrate that an exon skipping approach allows for the efficient excision of three previously untargeted *COL7A1* exons (31, 68, and 109). The *COL7A1* gene contains a repetitive collagenous domain comprised of 84 in‐frame exons,^27^ many of which may be amenable to therapeutic deletion. However, as several exons within this domain, including those within the “hinge” region, may be indispensable for protein function, C7 variants generated by exon deletion need to be assessed individually.^27^ To date, the therapeutic removal of several *COL7A1* exons within the collagenous domain (e.g. exons 70, 73, and 80), but also within the non‐collagenous domains (e.g., exons 13, 15, and 105), has been demonstrated primarily using antisense oligonucleotides.^17^, ^18^ Given the high proportion of pathogenic *COL7A1* variants that reside within protein coding regions, exon skipping represents a unified approach that could be tailored to address a high proportion of *COL7A1* mutations and therefore bypass the requirement for more expensive and complex personalized therapies. The research presented here uniquely focuses on pathogenic mutations present in three individuals from New Zealand and expands genotyping in this cohort. However, further work is required to determine if these are prevalent mutations in the NZ population affected by EB.^28^

Cas9‐mediated exon deletion has been reported twice for RDEB. In both cases a mutation in exon 80 was targeted, first by *in vivo* editing in an RDEB mouse model,^29^ and more recently *ex vivo* in primary RDEB keratinocytes.^7^ In our study, we employed a reframing strategy mediated by NHEJ repair, targeting three separate and previously untargeted *COL7A1* exons (31, 68, and 109). Exon skipping was achieved using nucleofection to deliver RNPs. This non‐viral approach has been shown to reduce off‐target effects *due to the short intracellular half‐life of the RNPs*, while still enabling robust on‐target editing.^20^, ^21^ At each locus, we routinely obtained exon deletion rates of at least 78%. These high editing efficiencies overcome some of the limitations associated with the lower correction rates obtained with many HDR‐based strategies. This includes bypassing the need for enrichment mechanisms and ensuring that a sufficient population of epidermal stem cells can be routinely targeted. Accordingly, the deletion of exon 31 in at least 78% of RDEB03 skin cells afforded the use of the entire polyclonal cell population for downstream analyses with no detectable off‐target activity at the top *in silico* predicted sites, as determined by Nanopore sequencing. The restoration of C7 expression as shown by immunofluorescence conferred the edited cells with adhesion properties similar to wild type cells *in vitro* and led to accurate C7 deposition at the dermal‐epidermal junction in a 3D skin model. Notably, the deletion of exon 31 proved to be highly targeted, resulting in the deletion of the intended fragment (±1–2 bp) in >60% of RDEB03 keratinocytes and >70% of RDEB03 fibroblasts. This was confirmed by analysis of *COL7A1* splicing which revealed that the majority of detected transcripts were spliced as expected.

We efficiently excised exon 31 from RDEB keratinocytes and fibroblasts. To the best of our knowledge, this is the first demonstration of exon skipping in dermal fibroblasts. Efficient protocols for editing both cell types are important as pre‐clinical evidence suggests that both may be necessary for generating and maintaining anchoring fibrils at the basement membrane.^30^ The addition of fibroblasts is also likely to provide beneficial wound healing benefits via the release of growth factors and extracellular matrix components.^31^ Moreover, analysis of RDEB fibroblasts has revealed aberrant expression patterns of genes involved in fibrosis, inflammation, and the development of squamous cell carcinomas.^32^ Therefore, the correction of RDEB fibroblasts may also help to reset the gene expression landscape to a physiological norm.

Structural variants generated by Cas9‐mediated genome editing have been detected at frequencies ranging from 0.5% to 40% across a range of human cell types.^33^ In addition to safety concerns regarding the potential genotoxic effects of SVs, such events can lead to inflated gene correction estimates and potentially mislead downstream analysis. In this study, using long‐read Nanopore sequencing, we detected large on‐target deletions spanning from several hundred bp to >5 kb in RDEB keratinocytes following exon 31 deletion. Strikingly, ~10% of these deletions evaded detection by short amplicon genotyping. These data highlight that standard short‐read sequencing techniques (e.g., Illumina) may underestimate the full range of editing outcomes, and therefore emphasizes the importance of performing more comprehensive genomic analysis to increase the accuracy of genotyping.

## 4 CONCLUSION

In this study, we report highly efficient and targeted dual Cas9‐RNP deletion of three separate *COL7A1* exons and show that the removal of exon 31 containing a pathogenic frameshift mutation restores the adhesion properties of RDEB fibroblasts, and leads to accurate C7 deposition to the basement membrane in a 3D skin model. The simplicity of RNP‐based delivery and the lack of requirement for positive selection mechanisms represents a simplified editing workflow which could enable a faster route into the clinic. As a predicted 107 out of 118 exons can be removed without disrupting the *COL7A1* open reading frame,^17^ exon skipping has a potentially broad applicability to address a high proportion of patient‐specific mutations. One limitation of the current study was the lack of *in vivo* experiments to assess C7 localization and dermal‐epidermal adhesion. Such experiments are a prerequisite for clinical translation to assess the long term efficacy of gene therapy strategies. Lastly, our research highlights the potential formation of SVs resulting from Cas9‐mediated genome editing. Therefore, we encourage researchers to incorporate long‐read sequencing into their editing workflows as it represents a relatively simple and fast method for assessing SV formation, allowing for a more accurate assessment of editing outcomes.

## 5 MATERIALS AND METHODS

### 5.1 Primary cell culture

Primary fibroblasts and keratinocytes, isolated from skin biopsies by whole‐skin digest,^34^ were taken from three fully informed and consented donors with RDEB (ethical approval number 19/STH/47 as provided by the Southern Health and Disability Ethics Committee). Commercially sourced primary dermal fibroblasts and human epidermal keratinocytes, adult (HEKa) were purchased from the American Type Culture Collection (ATCC).

Primary keratinocytes were cultured in Kelch's medium (DMEM:F12 3:1 [Gibco], 10% FBS [Moregate], 20 ng/mL KGF [Peprotech, Rocky Hill, NJ], 0.625 μg/mL amphotericin B [Sigma‐Aldrich], 100 U/mL PS, 0.4 μmol/L SB 772077B ROCK inhibitor [Tocris]).^34^ Primary fibroblasts were cultured in DF10 medium (DMEM, 10% FBS, 100 U/mL PS). Keratinocytes and fibroblasts were passaged when reaching 70%–90% and 80%–90% confluency, respectively. When passaging, culture medium was removed from culture flasks and the cells washed with Dulbecco's phosphate‐buffered saline (DPBS) (Gibco). Cells were detached with TrypLE (Gibco) for 3–5 min at 37°C with 5% CO_2_ and the TrypLE diluted out with growth medium. Dissociated cell suspensions were pelleted for 5 min at 400× *g*, resuspended in an appropriate growth medium and seeded into a new culture vessel at a density of 1 × 10^5^ cells/cm^2^.

### 5.2 Delivery of the CRISPR/Cas9 reagents into primary skin cells

Single guide RNAs (sgRNAs) were designed using a CRISPR design tool developed by Synthego Corporation (https://design.synthego.com/#/). Alt‐R™ S.p. Cas9 Nuclease V3 (IDT) and sgRNAs (Synthego) were delivered into primary skin cells as ribonucleoprotein (RNP) complexes using the Amaxa 4D Nucleofector (Lonza Bioscience). The P2 Primary Cell 4D Kit (Lonza Bioscience) was used for primary dermal fibroblasts and the P3 Primary Cell 4D Kit (Lonza Bioscience) for primary keratinocytes. Primary skin cells of passage 2 or 3 were detached with TrypLE, washed once with DPBS, and pelleted for 5 min at 400× *g*. 2 × 10^5^ cells per sample were resuspended in 20 μL of P2 or P3 nucleofection buffer (for dermal fibroblasts or keratinocytes, respectively) provided with each kit. RNP complexes were formed at room temperature (75 pmol sgRNA, 15.75 pmol Cas9 nuclease, 4.76:1 molar ratio) and added to the reactions immediately before nucleofection. The pulse programs CM‐137 and DT‐130 were used for primary keratinocytes and dermal fibroblasts, respectively. After nucleofection the cells were rested for 10–15 min at 37°C and then seeded into 6‐well plates with growth medium.

### 5.3 Nanopore library preparation and sequencing analysis

To generate Nanopore sequencing libraries, 200 fmol of DNA amplicon per sample was prepared using the Native Barcoding Kit 96 V14 (ONT, SQK‐NBD114.96). Prepared libraries were loaded onto flongle flow cells (FLO‐FLG114) and sequenced on a MinION sequencer (ONT, Mk1B) for 24 h. Guppy basecaller was used to generate Fastq files which were aligned to the human GRCh38 reference genome using minimap2 (version 2.24). Sequencing reads were then deconvoluted by selecting reads aligning to each locus and converting them back into Fastq format using SAMtools (version 1.15). For on‐target editing analysis, deconvoluted reads were first aligned to the GRCh38 reference genome using minimap2, and total editing calculated for each sgRNA using the CRISPRessoWGS tool. Deletions overlapping with predicted guide cleavage loci were identified and characterized using BEDtools (version 2.30.0). For off‐target amplicon analysis, deconvoluted reads were analyzed using the CRISPResso2 suite (version 2.2.11). Edited samples were compared to unedited control samples using the CRISPRessoCompare tool. For analysis of cDNA amplicons, deconvoluted reads were first aligned to the GRCh38 reference genome using minimap2 in splice aware mode using the intron‐exon junction from the primary *COL7A1* transcript (ID: ENST00000681320.1).

### 5.4 On‐target genotyping

Primary skin cells were prepared for genomic DNA (gDNA) extraction by centrifugation at 400× *g* for 5 min. Cell pellets were resuspended in 100 μL of cold DPBS containing 10 μL Liquid Proteinase K (Machery‐Nagel) and the gDNA extracted using the Monarch DNA Purification Kit (New England BioLabs). 50 ng of gDNA was used for PCR amplification using primers spanning the gRNA cut sites. Amplification was performed using a Phusion Hot Start II DNA Polymerase under the following thermal cycler settings: 1 min at 98°C; 30 cycles at 98°C for 10 s, 65°C for 30 s, 72°C for 40 s; and 72°C for 5 min. For long‐range PCR amplification the LongAmp *Taq* DNA Polymerase (New England BioLabs) was used under the following settings: 2 min at 94°C; 30 cycles at 94°C for 20 s, 60°C for 30 s, 65°C for 9.5 min; and 72°C for 10 min. All on‐target genotyping of edited skin cells was performed using gDNA extracted 3 days post‐editing.

### 5.5 Off‐target analysis

The top in silico predicted off‐target sites were selected for analysis using the Synthego gRNA validation tool (https://design.synthego.com/#/validate). Off‐target sites were amplified by PCR and Nanopore sequenced (Oxford Nanopore Technologies, as described above). Off‐target genotyping of edited skin cells was performed using gDNA extracted 3 days post‐editing.

### 5.6 *COL7A1* mRNA analysis using ddPCR and RT‐PCR

Total RNA was extracted from primary fibroblasts and keratinocytes using the RNAqueous Total RNA Isolation Kit (Thermo Fisher Scientific) and used as a template to synthesize cDNA using the SuperScript III First‐Strand Synthesis kit (Invitrogen). Quantification of *COL7A1* transcription was conducted using the QX200 ddPCR system (Bio‐Rad) and was based on the fluorescence of the nucleic acid binding dye EvaGreen. The expression of the housekeeping genes TBP, HPRT‐1, and GAPDH were used for normalization. QuantaSoft Analysis Pro was used for data analysis.

To analyze *COL7A1* splicing, exons 28–40 of *COL7A1* cDNA were amplified under the following thermal cycler settings: 1 min at 98°C; 30 cycles of 98°C for 10 s, 65°C for 30 s, 72°C for 40 s; and 72°C for 5 min. Amplicons were subject to Nanopore sequencing, as described above.

### 5.7 Protein extraction and Western Blot analysis

Primary keratinocytes were lysed in radioimmunoprecipitation assay (RIPA) buffer (Thermo Fisher Scientific) containing 1% Halt Protease Inhibitor Cocktail (Thermo Fisher Scientific) and incubated on ice for 30 min followed by centrifugation for 15 min at 14,000× *g* at 4°C. Supernatants containing total protein were quantified using the Pierce BCA Protein Assay Kit (Thermo Fisher Scientific). 40 μg total protein was resolved via gel electrophoresis using NuPage 3%–8% Tris‐Acetate gels (Invitrogen) and subsequently transferred to nitrocellulose membranes (Bio‐Rad). Membranes were stained with Ponceau S (Thermo Fisher Scientific) to assess protein transfer. To analyze C7, blots were probed with a monoclonal anti‐C7 antibody (Clone LH7.2; Abcam, ab6312). GAPDH was used as a loading control using a monoclonal anti‐GAPDH antibody (Clone 6C5; Abcam, ab8245). For detection, blots were probed with an IRDye 800CW donkey anti‐Mouse secondary antibody (Li‐Cor Biosciences) and then imaged using the Odyssey CLx imaging system (Li‐Cor Biosciences).

### 5.8 Immunofluorescence of skin and primary keratinocytes

For immunofluorescent analysis of primary keratinocytes, cells were seeded into 96‐well plates (Thermo Fisher Scientific) and cultured for 3 days. Cells were fixed with 4% paraformaldehyde (Thermo Fisher Scientific) for 15 min at room temperature followed by permeabilization with 0.5% Triton X‐100 (Sigma Life Science) for 15 min at room temperature. Following 3 × 10 min washes with PBS, cells were blocked with a 0.25% casein solution in TBS containing 10% human serum (HS) (Thermo Fisher Scientific) for 10 min at room temperature. The following antibodies were used for primary detection: mouse monoclonal IgG1 anti‐C7 (Clone LH7.2; Abcam, ab6312) at a 1:200 dilution, mouse monoclonal IgG1 anti‐CK14 (Clone RCK107, Abcam, ab9220) at a 1:200 dilution, rabbit monoclonal anti‐Ki‐67 (Clone SP6, Abcam, ab16667) at a 1:500 dilution. Primary antibody cocktails were diluted in dilution buffer (DB) (10% HS in TBS) and applied overnight at 4°C. Following 3 × 10‐min washes with TBS, secondary detection was performed using the following antibodies diluted in DB: Goat anti‐mouse IgG1 Alexa Fluor 647 (Molecular Probes, #A21240) at a 1:200 dilution, and goat anti‐rabbit IgG Alexa Fluor 555 (Molecular Probes, #A21428) at a 1:1000 dilution. DAPI was used as a nuclear stain at a final concentration of 2.5 μg/mL. Secondary antibody cocktails were applied for 30 min at room temperature. The Nikon Ni‐U Upright fluorescent microscope was used for imaging. ImageJ was used for quantification of Ki‐67 or C7‐positive keratinocytes.

For immunofluorescent staining of cross sections from a 3D skin model or skin sections from RDEB and healthy donors, glass slides containing 5 μm skin sections were subject to the same process as outlined above. In addition to the antibodies mentioned above, a rabbit IgG anti‐Claudin‐1 (Clone EPRR18871; Abcam, ab211737) at a 1:300 dilution and a goat anti‐rabbit IgG Alexa Fluor 555 (Molecular Probes, #A21428) at a 1:1000 dilution was used as an epidermal stain to detect Claudin‐1. ProLong Gold Antifade reagent was applied to the stained slides which were subsequently mounted with cover slips. The Nikon Ni‐U Upright fluorescent microscope was used for imaging.

### 5.9 Generation of a 3D skin model

To construct the 3D skin model, a synthetic copolymer poly(lactic‐co‐glycolic acid) (PLGA) mesh was used as a dermal scaffold. A 50 μg/mL type I collagen solution containing 6 mg/mL acid soluble collagen in 0.01 M HCI (Collagen Solutions) diluted with H_2_0, 0.1 M NaOH and PBS was used to coat one side of the mesh for 1 h at 37°C. The collagen coated side of the mesh was washed twice with PBS and seeded with 1 × 10^5^ fibroblasts/cm^2^. 1 cm^2^ pieces of mesh were incubated in 12‐well plates submerged in DF10 medium for 4 h at 37°C (5% CO_2_) to allow fibroblast adherence. The opposite side of the mesh was then seeded with 3 × 10^5^ keratinocytes and incubated overnight at 37°C (5% CO_2_) submerged in Kelch's medium without the SB 772077B ROCK inhibitor. The following day the samples were raised to an air‐liquid interface and cultured for 3 weeks in Kelch's medium without the ROCK inhibitor to form a stratified epithelium. After 3 weeks, the samples were embedded in Tissue Plus O.C.T embedding matrix (Scigen) and frozen at −80°C. 5 μm sections were cut using a Leica CM1860UV cryotome for immunofluorescence staining.

### 5.10 In vitro adhesion assay of primary fibroblasts

For analysis of skin cell adhesion in vitro, 1 × 10^4^ primary fibroblasts were plated in 96‐well plates and cultured for 24 h. Cells were washed twice with DPBS and then incubated with the TrypLE dissociation reagent for different timepoints (0, 1, 2, 4, 6 min). Following two more washes with DPBS, the cells were fixed and stained with 0.5% crystal violet (containing 10% methanol) (Sigma‐Aldrich) for 10 min at room temperature. Following two more DPBS washes, the cells were solubilized by incubating with a 1% sodium dodecyl sulfate (SDS) solution (Sigma‐Aldrich) for 1 h. Absorbance at 590 nm was measured to determine the percentage of adherent cells.

### 5.11 Statistical analysis

Statistical analysis was performed using R studio (version 2021.09.0). Two‐sample *t*‐tests were used to determine statistically significant differences between test and control samples. Different levels of significance were denoted with *p*‐values <0.05, <0.01, or <0.001. Data are represented as the mean ± the standard error of the mean.

## AUTHOR CONTRIBUTIONS

**Alex du Rand:** Formal analysis (lead); investigation (lead); methodology (lead); writing – original draft (lead). **John Hunt:** Methodology (supporting); writing – review and editing (supporting). **Christopher Samson:** Formal analysis (supporting); methodology (supporting). **Evert Loef:** Methodology (supporting). **Chloe Malhi:** Methodology (supporting). **Sarah Meidinger:** Methodology (supporting). **Chun‐Jen Jennifer Chen:** Methodology (supporting). **Ashley Nutsford:** Methodology (supporting). **John Taylor:** Resources (supporting). **P. Rod Dunbar:** Resources (supporting); writing – review and editing (supporting). **Diana Purvis:** Resources (supporting); writing – review and editing (supporting). **Vaughan Feisst:** Methodology (supporting); writing – review and editing (supporting). **Hilary Sheppard:** Conceptualization (lead); funding acquisition (lead); project administration (lead); supervision (lead); writing – review and editing (lead).

## FUNDING INFORMATION

This work was supported by grants from the Auckland Medical Research Foundation (AMRF), the Faculty of Science (FoS) Research Development Fund (the University of Auckland), CureKids, the School of Biological Sciences (the University of Auckland), and the Maurice Phyliss Paykel Trust.

## CONFLICT OF INTEREST STATEMENT

The authors declare no competing interests.

### PEER REVIEW

The peer review history for this article is available at https://www.webofscience.com/api/gateway/wos/peer-review/10.1002/btm2.10640.

## Supporting information


**FIGURE S1:** Immunocytochemistry of the healthy keratinocytes and RDEB03 keratinocytes used in this study. The keratinocyte‐specific marker cytokeratin 14 (CK14) is stained in green and used to confirm the keratinocyte lineage. DAPI was used as a nuclear stain. Scale bars = 50 μm.
**FIGURE S2:** Proliferation analysis of RDEB03 keratinocytes following dual Cas9‐RNP exon skipping compared to untreated RDEB03 keratinocytes and wild type keratinocytes. (a) Representative immunofluorescence images showing the proliferation marker Ki‐67 in RDEB03 keratinocytes (left), exon skipped RDEB03 keratinocytes (middle) and wild type keratinocytes (right). Imaging was performed 6 days after dual Cas9‐RNP editing. A pair‐wise *T* test (*p* = 0.05) between the samples (*n* = 3 per sample) found no statistical difference in Ki‐67 expression between the three keratinocyte populations. Scale bars = 50 μm. (b) Proliferation curve comparing the number of population doublings between RDEB03 keratinocytes, exon skipped RDEB03 keratinocytes and wild type keratinocytes over a 26 day cell expansion period. Dual Cas9‐RNP editing of RDEB03 keratinocytes was performed at day zero. Individual datapoints represent cell counts. Data are representative of two separate keratinocyte expansions.
**FIGURE S3:** Nanopore sequencing analysis of the editing outcomes resulting from dual Cas9 nickase RNP exon skipping in primary keratinocytes. (a) Diagrammatic representation of the 10 most common deletion events resulting from dual Cas9 nickase RNP editing in primary keratinocytes. The wild type allele is shown for each locus. The position of the sgRNAs is also shown, with the PAM sites underlined. The predicted Cas9 cut sites are depicted with red dotted lines. For each of the edited alleles, the allele frequency and the deletion size are shown. (b) Summary of the total editing and exon deletion efficiencies for each *COL7A1* exon.
**FIGURE S4:** Analysis of wild type keratinocytes following exon 68 or 109 deletion compared to unedited keratinocytes. (A) Immunofluorescence of wild type keratinocytes following exon 68 deletion (left), exon 109 deletion (middle) or unedited keratinocytes (right). C7 is shown in green and DAPI in blue. Exon deletion percentages correspond to the proportion of alleles with a deletion spanning the target exon. Scale bars = 50 μm. (b) The proportion of C7‐positive keratinocytes between the three populations. Data represent the mean ± SEM, *n* = 3. ns, no significance.
**TABLE S1:** List of guide RNA (gRNA) sequences.
**TABLE S2:** List of PCR primer sets used for short and long‐range on‐target genotyping, off‐target genotyping and mRNA analysis.
**TABLE S3:** Off‐target analysis at the top in silico predicted off‐target sites for the sgRNA pair targeting exon 31 of *COL7A1* generated by CRISPResso2.
**TABLE S4:** Number of Nanopore sequencing read numbers analyzed for *COL7A1* splicing analysis.
**TABLE S5:** Frequency (%) of *COL7A1* splicing isoforms detected by Nanopore sequencing of amplicons spanning exons 28–41 in *COL7A1* cDNA.

## Data Availability

The data from this study are available from the corresponding author on reasonable request.
