# Supplementary material for: Highly efficient CRISPR/Cas9‐mediated exon skipping for recessive dystrophic epidermolysis bullosa
Source: Bioeng Transl Med. 2024 Jan 17;9(4):e10640. doi: 10.1002/btm2.10640 (PMC11256143; doi:10.1002/btm2.10640)
Supplement: Supplementary file 1 — FIGURE S1: Immunocytochemistry of the healthy keratinocytes and RDEB03 keratinocytes used in this study. The keratinocyte‐specific marker cytokeratin 14 (CK14) is stained in green and used to confirm the keratinocyte lineage. DAPI was used as a nuclear stain. Scale bars = 50 μm. FIGURE S2: Proliferation analysis of RDEB03 keratinocytes following dual Cas9‐RNP exon skipping compared to untreated RDEB03 keratinocytes and wild type keratinocytes. (a) Representative immunofluorescence images showing the proliferation marker Ki‐67 in RDEB03 keratinocytes (left), exon skipped RDEB03 keratinocytes (middle) and wild type keratinocytes (right). Imaging was performed 6 days after dual Cas9‐RNP editing. A pair‐wise T test (p = 0.05) between the samples (n = 3 per sample) found no statistical difference in Ki‐67 expression between the three keratinocyte populations. Scale bars = 50 μm. (b) Proliferation curve comparing the number of population doublings between RDEB03 keratinocytes, exon skipped RDEB03 keratinocytes and wild type keratinocytes over a 26 day cell expansion period. Dual Cas9‐RNP editing of RDEB03 keratinocytes was performed at day zero. Individual datapoints represent cell counts. Data are representative of two separate keratinocyte expansions. FIGURE S3: Nanopore sequencing analysis of the editing outcomes resulting from dual Cas9 nickase RNP exon skipping in primary keratinocytes. (a) Diagrammatic representation of the 10 most common deletion events resulting from dual Cas9 nickase RNP editing in primary keratinocytes. The wild type allele is shown for each locus. The position of the sgRNAs is also shown, with the PAM sites underlined. The predicted Cas9 cut sites are depicted with red dotted lines. For each of the edited alleles, the allele frequency and the deletion size are shown. (b) Summary of the total editing and exon deletion efficiencies for each COL7A1 exon. FIGURE S4: Analysis of wild type keratinocytes following exon 68 or 109 deletion compared to u [file BTM2-9-e10640-s001.docx]

**Highly efficient CRISPR/Cas9-mediated exon skipping for recessive dystrophic epidermolysis bullosa**

**The Journal of Bioengineering & Translational Medicine**

Alex du Rand, John Hunt, Christopher Samson, Evert Loef, Chloe Malhi, Sarah Meidinger, Chun-Jen Jennifer Chen, Ashley Nutsford, John Taylor, Rod Dunbar, Diana Purvis, Vaughan Feisst, Hilary Sheppard

Corresponding author: Hilary Sheppard ([h.sheppard@auckland.ac.nz](mailto:h.sheppard@auckland.ac.nz)), the School of Biological Sciences, the University of Auckland, Auckland, New Zealand.

**Fig. S1**


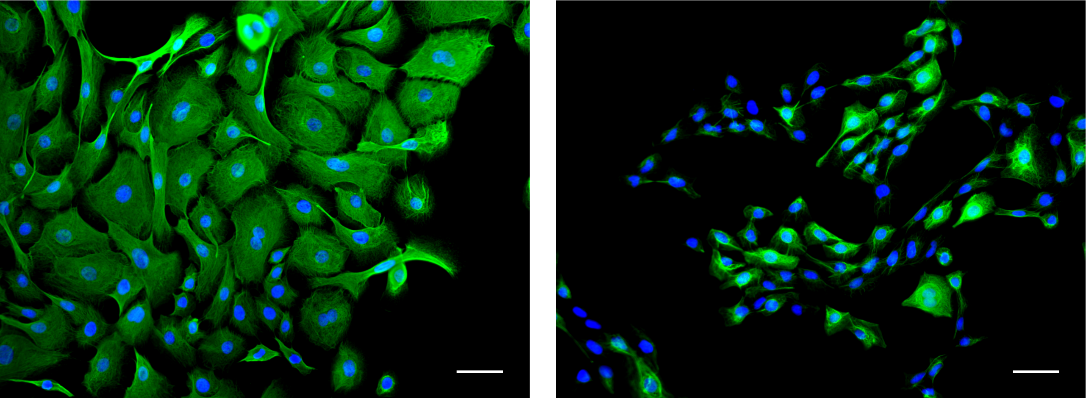


RDEB03 keratinocytes

Healthy keratinocytes

**DAPI** **CK14**

Immunocytochemistry of the healthy keratinocytes and RDEB03 keratinocytes used in this study. The keratinocyte-specific marker cytokeratin 14 (CK14) is stained in green and used to confirm the keratinocyte lineage. DAPI was used as a nuclear stain. Scale bars = 50 µm.

**Fig. S2**

Proliferation analysis of RDEB03 keratinocytes following dual Cas9-RNP exon skipping compared to untreated RDEB03 keratinocytes and wild type keratinocytes. (A) Representative immunofluorescence images showing the proliferation marker Ki-67 in RDEB03 keratinocytes (left), exon skipped RDEB03 keratinocytes (middle) and wild type keratinocytes (right). Imaging was performed 6 days after dual Cas9-RNP editing. A pair-wise T test (p=0.05) between the samples (n=3 per sample) found no statistical difference in Ki-67 expression between the three keratinocyte populations. Scale bars = 50 µm. (B) Proliferation curve comparing the number of population doublings between RDEB03 keratinocytes, exon skipped RDEB03 keratinocytes and wild type keratinocytes over a 26 day cell expansion period. Dual Cas9-RNP editing of RDEB03 keratinocytes was performed at day zero. Individual datapoints represent cell counts. Data is representative of two separate keratinocyte expansions.


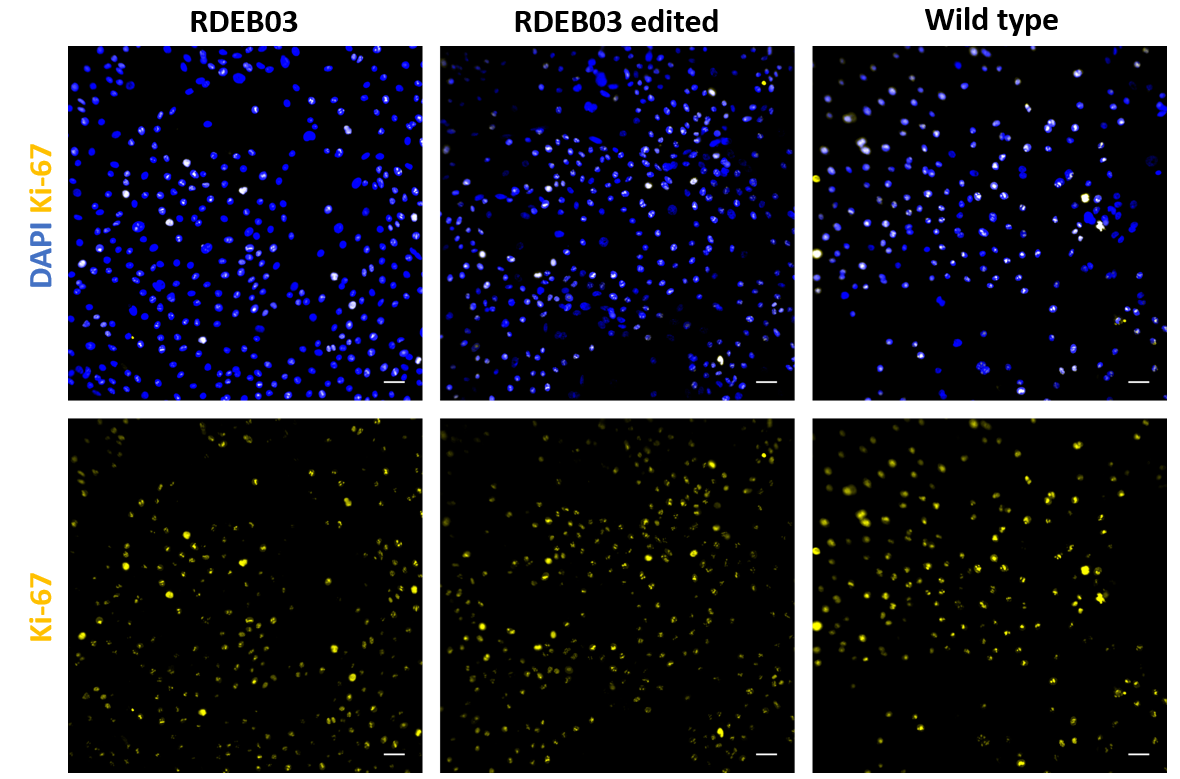

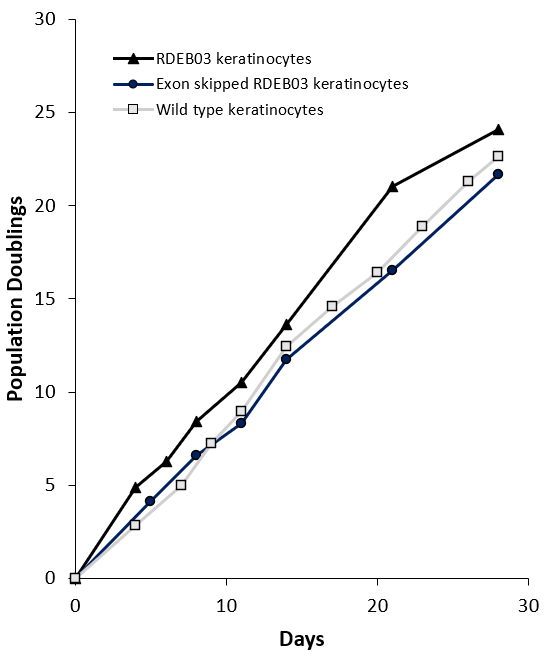


**Fig. S3**

**Dual Cas9 nickase - wild type keratinocytes (exon 109)**

**Dual Cas9 nickase - wild type keratinocytes (exon 68)**

**Dual Cas9 nickase - wild type keratinocytes (exon 31)**


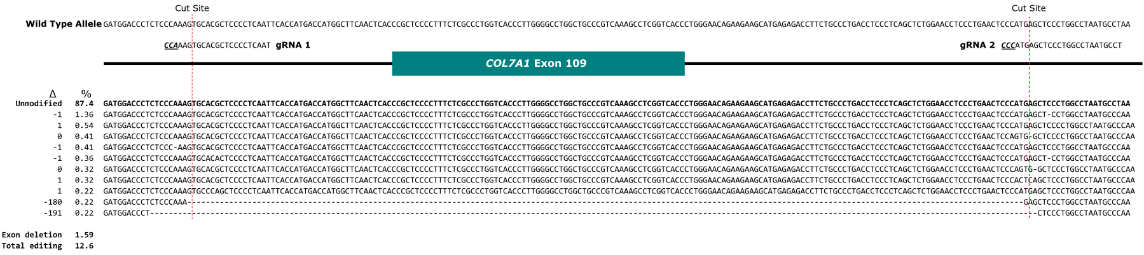

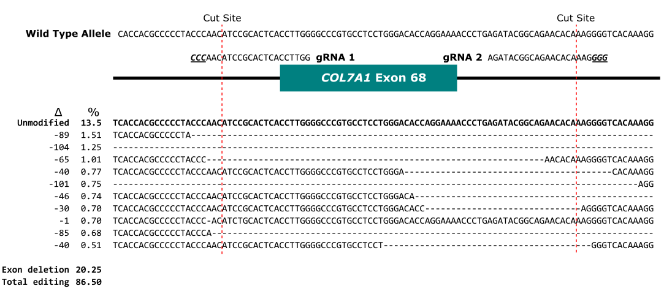

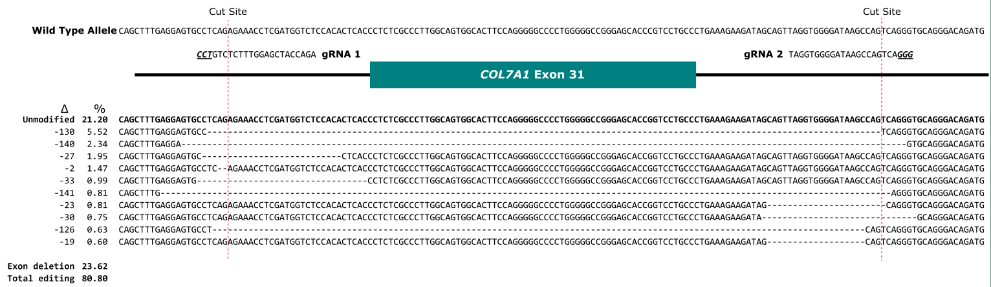


**A**

**Dual Cas9 nickase - RDEB03 keratinocytes (exon 31)**

***
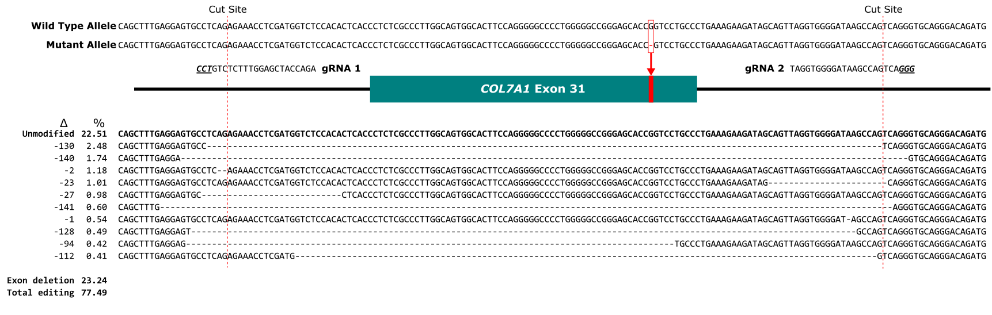
***

**B**

| **Donor** | **Exon** | **Editing (%)** | **Exon deletion (%)** |
| --- | --- | --- | --- |
| Wild type keratinocytes | 109 | 12.60 | 1.59 |
| Wild type keratinocytes | 68 | 86.50 | 20.25 |
| Wild type keratinocytes | 31 | 80.80 | 23.62 |
| RDEB03 keratinocytes | 31 | 77.49 | 23.24 |

Nanopore sequencing analysis of the editing outcomes resulting from dual Cas9 nickase RNP exon skipping in primary keratinocytes. (A) Diagrammatic representation of the 10 most common deletion events resulting from dual Cas9 nickase RNP editing in primary keratinocytes. The wild type allele is shown for each locus. The position of the sgRNAs is also shown, with the PAM sites underlined. The predicted Cas9 cut sites are depicted with red dotted lines. For each of the edited alleles, the allele frequency and the deletion size are shown. (B) Summary of the total editing and exon deletion efficiencies for each *COL7A1* exon.

**Fig. S4**


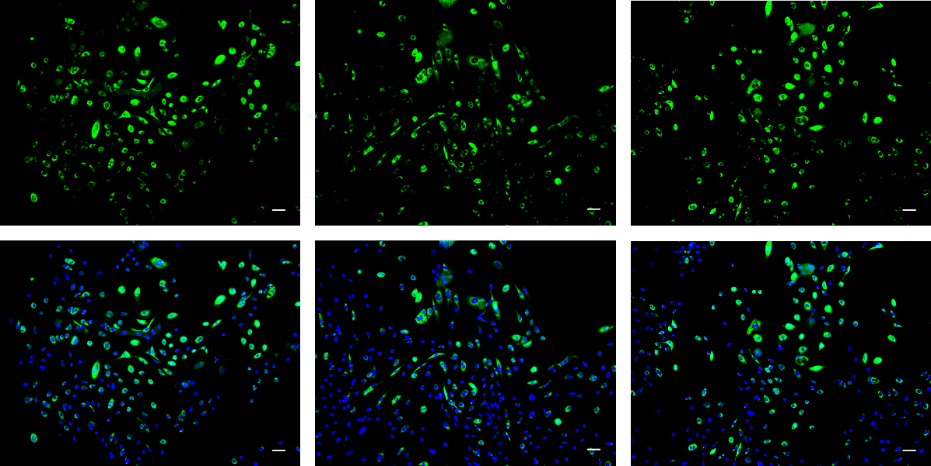


**95% exon 68 deletion**

**90% exon 109 deletion**

**Unedited**

**DAPI C7**

**C7**


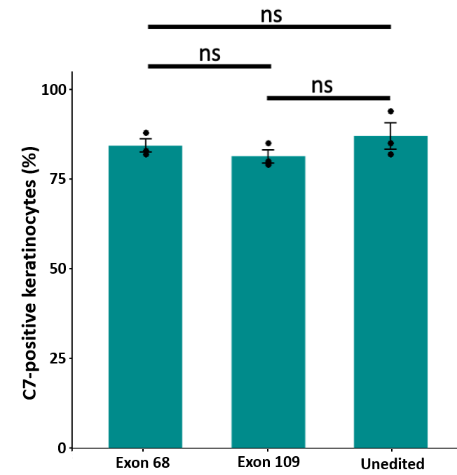


Analysis of wild type keratinocytes following exon 68 or 109 deletion compared to unedited keratinocytes. (A) Immunofluorescence of wild type keratinocytes following exon 68 deletion (left), exon 109 deletion (middle) or unedited keratinocytes (right). C7 is shown in green and DAPI in blue. Exon deletion percentages correspond to the proportion of alleles with a deletion spanning the target exon. Scale bars = 50 µm. (B) The proportion of C7-positive keratinocytes between the three populations. Data represent the mean ± SEM, n = 3. ns, no significance

**A**

**B**

**Table S1** List of guide RNA (gRNA) sequences

| **Name** | **Target** | **Sequence** |
| --- | --- | --- |
| Exon 31, gRNA1 | *COL7A1*, intron 30 | UAGGUGGGGAUAAGCCAGUC-AGG |
| Exon 31, gRNA2 | *COL7A1*, intron 31 | GAUAAGCCAGUCAGGGUGCA-GGG |
| Exon 68, gRNA1 | *COL7A1*, intron 67 | CUUUGUGUUCUGCCGUAUCU-GGG |
| Exon 68, gRNA2 | *COL7A1*, intron 68 | CCAAGGUGAGUGCGGAUGUU-GGG |
| Exon 109, gRNA1 | *COL7A1*, intron 108 | CAUUAGGCCAGGGAGCUCAU-GGG |
| Exon 109, gRNA2 | *COL7A1*, intron 109 | AUUGAGGGGAGCGUGCACUU-TGG |

PAM sites are indicated in red.

**Table S2** List of PCR primer sets used for short and long range on-target genotyping, off-target genotyping and mRNA analysis

| **Name** | **Analysis** | **Target** | **Sequence (5’-3’)** |
| --- | --- | --- | --- |
| Exon 31 | On-target genotyping | *COL7A1*, Exon 31 | **FWD**: CCTGTGTCAGGCATCCTTCA  **REV**: GTCCCCCAAAGTCTCTGTGG |
| Exon 31 Long Range | Long Range On-target genotyping | *COL7A1*, Exon 31 | **FWD**: TACCTCCAGCCCTCTCCTTC  **REV**: TACTCCCTTACCCGCCATGA |
| Exon 68 | On-target genotyping | *COL7A1*, Exon 68 | **FWD**: GCAGGCACACCCTTAGACAT  **REV**: TTGCCCAGAGCACCCTTTAG |
| Exon 109 | On-target genotyping | *COL7A1*, Exon 109 | **FWD**: GGTGTAGGGACAAGGAAGGC  **REV**: GTCAGTGCAGCTTCTCCCTT |
| Exon 31, sgRNA1, Off-Target 1 | Off-target analysis | PLPP1, Chr 5 | **FWD**: CAGCTCGCTCCAACACTTCTA  **REV**: CAGCAGGTCTCTGGGTGATG |
| Exon 31, sgRNA1, Off-Target 2 | Off-target analysis | Intergenic, Chr 8 | **FWD**: TGAGATTTGCGTTGTCCCCT  **REV**: TCTTACAGCGAGTAAGCCACG |
| Exon 31, sgRNA1, Off-Target 3 | Off-target analysis | Intergenic, Chr 3 | **FWD**: GGGGACTAGACCAACATTAGCC  **REV**: CTCACATGGGAACCATATTCCAC |
| Exon 31, sgRNA1, Off-Target 4 | Off-target analysis | Intergenic, Chr 10 | **FWD**: TCATATGCGGATCATGCCGA  **REV**: GGCATAGTGGTCTTTTGTTTGGT |
| Exon 31, sgRNA1, Off-Target 5 | Off-target analysis | *ABLIM2*, Chr 4 | **FWD**: CCAATCACAAGCACGGATGG  **REV**: ATGTGTGTGCTGACAAGCGG |
| Exon 31, sgRNA1, Off-Target 6 | Off-target analysis | *WDR1*, Chr 4 | **FWD**: CATCACTGAGACGGCGAAGA  **REV**: CACATCTGGGAGGACCGAAG |
| Exon 31, sgRNA2, Off-Target 1 | Off-target analysis | Intergenic, Chr 3 | **FWD**: TGGAGTCAGCAGTCCTTTGC  **REV**: CAGCAGAACCGACAAGGCTA |
| Exon 31, sgRNA2, Off-Target 2 | Off-target analysis | *ANK1*, Chr 8 | **FWD**: TAGCATGGTGGCGGGTACAA  **REV**: ATGTCCAGATTGCCTGGTGC |
| Exon 31, sgRNA2, Off-Target 3 | Off-target analysis | *GSE1*, Chr 16 | **FWD**: TGGAGGCAGGGGACTTCTAC  **REV**: AAGAGACCCTCATTCTCCGAAC |
| Exon 31, sgRNA2, Off-Target 4 | Off-target analysis | *DPPA2P2*, Chr 1 | **FWD**: CACCAGCGGCTTGCATCAAA  **REV**: TGACTGTGGTCAGGGGATGAAT |
| Exon 31, sgRNA2, Off-Target 5 | Off-target analysis | *TARID*, Chr 6 | **FWD**: TAGTTGCTTCTTAAGCTGCCC  **REV**: GGCAGTTATAGTTACGCAGGC |
| Exon 31, sgRNA2, Off-Target 6 | Off-target analysis | *LAMB3,* Chr 1 | **FWD**: CCCACCGCAGTTCAAAAGC  **REV**: CCTCCTCTCTAATACGCGCAG |
| *COL7A1* splicing primers | *COL7A1* splicing analysis | Exon 28-40, *COL7A1* cDNA | **FWD**: CCTTCTTCGCCGTGGATGAT  **REV**: GAGCTATTGGCCCCAAA |
| *COL7A1* ddPCR primers | *COL7A1* quantification | Exon 109, *COL7A1* cDNA | **FWD**: GTGACCAGGGCGAGAAA  **REV**: CAGCAGAGCCATCATTTCCA |
| ddPCR housekeeper, GAPDH | *COL7A1* quantification | *GAPDH* | **FWD**: CCAAGGTCATCCATGACAACT  **REV**: GGCCATCCACAGTCTTCTG |
| ddPCR housekeeper, HPRT-1 | *COL7A1* quantification | *HPRT-1* | **FWD**: GCTGAGGATTTGGAAAGGGT  **REV**: CCTTCATCACATCTCGAGCAA |
| ddPCR housekeeper, TBP | *COL7A1* quantification | *TBP* | **FWD**: GAGTTCTGGGATTGTACCGC  **REV**: CACGAAGTGCAATGGTCTTT |

**Table S3** Off-target analysis at the top *in silico* predicted off-target sites for the sgRNA pair targeting exon 31 of *COL7A1* generated by CRISPResso2

| **Off-target site** | **Control read number** | **Edited RDEB03 fibroblast read number** | **Min. p-value** | **Min p-value Bonferroni Corrected** | **Control read number** | **Edited RDEB03 keratinocyte read number** | **Min. p-value** | **Min p-value Bonferroni Corrected** |
| --- | --- | --- | --- | --- | --- | --- | --- | --- |
| sgRNA1 Site 1 | 17263 | 36881 | 0.185 | 1 | 17263 | 30413 | 0.020 | 1 |
| sgRNA1 Site 2 | 10847 | 19239 | 0.138 | 1 | 10847 | 14290 | 0.247 | 1 |
| sgRNA1 Site 3 | 14845 | 27196 | 0.465 | 1 | 14845 | 25826 | 0.388 | 1 |
| sgRNA1 Site 4 | 10202 | 16379 | 0.079 | 1 | 10202 | 14559 | 0.056 | 1 |
| sgRNA1 Site 5 | 13208 | 17771 | 0.518 | 1 | 13208 | 15270 | 0.397 | 1 |
| sgRNA1 Site 6 | 10993 | 25051 | 0.062 | 1 | 10993 | 19722 | 0.018 | 1 |
| sgRNA2 Site 1 | 34880 | 25823 | 0.181 | 1 | 34880 | 23889 | 0.121 | 1 |
| sgRNA2 Site 2 | 16799 | 29186 | 0.059 | 1 | 16799 | 88303 | 0.005 | 0.495 |
| sgRNA2 Site 3 | 9983 | 5901 | 0.085 | 1 | 9983 | 7159 | 0.340 | 1 |
| sgRNA2 Site 4 | 34981 | 20531 | 0.074 | 1 | 34981 | 68861 | 0.040 | 1 |
| sgRNA2 Site 5 | 21362 | 12830 | 0.023 | 1 | 21362 | 14617 | 0.009 | 0.853 |
| sgRNA2 Site 6 | 17520 | 15744 | 0.019 | 1 | 17520 | 14017 | 0.097 | 1 |

**Table S4:** Number of Nanopore sequencing read numbers analyzed for *COL7A1* splicing analysis

| **Sample** | **Read number** |
| --- | --- |
| RDEB03 fibroblasts exon skipped | 68051 |
| RDEB03 fibroblasts control | 70531 |
| RDEB03 keratinocytes exon skipped | 87684 |
| RDEB03 keratinocytes control | 44881 |
| Wild type fibroblasts | 70547 |
| Wild keratinocytes | 79467 |

**Table S5** Frequency (%) of *COL7A1* splicing isoforms detected by Nanopore sequencing of amplicons spanning exons 28-41 in *COL7A1* cDNA

| **Transcript** | **Wild type keratinocytes (%)** | **Unedited RDEB03 keratinocytes (%)** | **Edited RDEB03 keratinocytes (%)** | **Wild type fibroblasts (%)** | **Unedited RDEB03 fibroblasts (%)** | **Edited RDEB03 Fibroblasts (%)** |
| --- | --- | --- | --- | --- | --- | --- |
| **Exon 29 Skip** | 0.18 | 0.14 | 0.00 | 0.14 | 0.11 | 0.01 |
| **Exon 29-31 Skip** | 0.07 | 0.09 | 0.35 | 0.15 | 0.17 | 0.01 |
| **Exon 30 Skip** | 0.01 | 0.02 | 2.30 | 0.03 | 0.01 | 0.35 |
| **Exon 30-31 Skip** | 0.03 | 0.05 | 1.25 | 0.02 | 0.01 | 0.04 |
| **Exon 31 & 36 Skip** | 0.00 | 0.00 | 0.19 | 0.00 | 0.00 | 0.14 |
| **Exon 31 & 36-37 Skip** | 0.00 | 0.00 | 0.08 | 0.00 | 0.00 | 0.25 |
| **Exon 31 & 37-39 Skip** | 0.00 | 0.00 | 0.23 | 0.00 | 0.00 | 0.96 |
| **Exon 31 & 38 Skip** | 0.00 | 0.00 | 1.00 | 0.00 | 0.00 | 0.04 |
| **Exon 31 Skip** | 0.03 | 0.04 | 88.65 | 0.03 | 0.03 | 84.91 |
| **Exon 31 Skip & Novel Exon** | 0.00 | 0.00 | 0.64 | 0.00 | 0.00 | 0.29 |
| **Exon 32-39 Skip** | 0.00 | 0.02 | 0.18 | 0.00 | 0.00 | 7.87 |
| **Exon 32-36 Skip** | 0.08 | 0.01 | 0.00 | 0.14 | 0.01 | 0.00 |
| **Exon 33-34 Skip** | 0.03 | 0.08 | 0.00 | 0.13 | 0.09 | 0.01 |
| **Exon 36 Skip** | 0.19 | 0.16 | 0.02 | 0.14 | 0.18 | 0.01 |
| **Exon 36-37 Skip** | 0.33 | 0.07 | 0.00 | 0.24 | 0.12 | 0.02 |
| **Exon 37 Skip** | 0.22 | 0.28 | 0.01 | 0.29 | 0.29 | 0.02 |
| **Exon 37-39 Skip** | 0.99 | 0.86 | 0.00 | 1.61 | 0.82 | 0.04 |
| **Exon 38 Skip** | 0.06 | 0.61 | 0.05 | 0.30 | 0.08 | 0.00 |
| **Full Transcript** | 97.01 | 96.68 | 3.33 | 96.09 | 97.54 | 4.07 |
